# United Network for Organ Sharing (UNOS) Database Analysis of Factors Associated With Kidney Transplant Time on Waiting List

**DOI:** 10.7759/cureus.34679

**Published:** 2023-02-06

**Authors:** Kristina Fritz, Jennifer Hong, Devina Basdeo, Kimberly Byrnes, Andres Cordoba, Kylie Dunn, Umbul Haider, Mareena Kashif, Nick Lee, Aysha S Mohamed Nuhuman, Radleigh Santos, Robin J Jacobs

**Affiliations:** 1 Medicine, Dr. Kiran C. Patel College of Osteopathic Medicine, Nova Southeastern University, Fort Lauderdale, USA; 2 Mathematics, Nova Southeastern University, Davie, USA; 3 Epidemiology and Public Health, Dr. Kiran C. Patel College of Osteopathic Medicine, Nova Southeastern University, Fort Lauderdale, USA

**Keywords:** minority donors, asian, african americans, blacks, racial minorities, health disparity, wait list, organ procurement, kidney transplantation

## Abstract

Introduction: In the United States (U.S.), African Americans and other minority groups have longer wait times for kidney transplantation than Caucasians. To date, many studies analyzing time spent on the waitlist for each race/ethnicity have been done. However, there are few to no studies examining waitlist time after the 2019 policy changes to the geographic distribution of donated kidneys.

Methods: Data collected from the National Organ Procurement and Transplantation Network database were used to analyze associations between race and time spent on the waitlist for a kidney transplant in the U.S. Additional sub-categorical data were analyzed to determine further associations and potential covariates, such as gender, age, citizenship, primary source of payment, region of transplant center, BMI, Kidney Donor Profile Index (KDPI), renal diagnosis, and presence/type of diabetes. Data were analyzed using odds ratios and validated by Bonferroni-Holm’s corrected chi-square tests at confidence intervals of 95% to determine if there are statistically significant differences between transplant time spent on the waitlist and ethnicity, as well as age, diagnosis category, region of transplant center, and KDPI.

Results: Statistically significant increased odds of remaining on the transplant list at two years existed for all non-white races/ethnicities, except those identifying as multiracial. Asian American candidates had the greatest odds of remaining on the waitlist greater than two years in comparison to white candidates: 1.51 times that of a patient categorized as white (odds ratio [OR] 1.51, confidence interval [CI] 1.44-1.57). African American/Black, (OR 1.38, CI 1.34-1.43) Pacific Islander (OR 1.38, CI 1.17-1.63), Hispanic candidates (OR 1.37, CI 1.32-1.41), and American Indian or Native Alaskan candidates (OR 1.23, CI 1.12-1.46) also had increased odds of remaining on the transplant waitlist greater than two years compared to white candidates.

Discussion: In this study, ethnic disparities persisted as a barrier for non-white individuals receiving treatment for end-stage kidney disease, specifically in the context of time spent on the waitlist for a kidney transplant. Further research is needed regarding the causes of these disparities in time spent on the waitlist, such as cultural restrictions in organ donation, racial differences in parameters for organ match, and institutionalized racism in health care practitioners.

## Introduction

In 2022, the United States (U.S.) organ transplant system achieved its one-millionth transplant, a milestone in organ transplant history [1]. The United Network for Organ Sharing (UNOS) serves as the nation’s transplant system under contract with the federal government. While organ procurement and transplants have become more streamlined, there are specific populations that have a more difficult time securing a transplantable organ, such as a kidney. Kidney transplant patients are the largest population of organ transplant recipients and the largest population of those remaining on the organ transplant waitlist. In 2021, more than 24,000 people received a kidney transplant out of the 41,354 organ transplants. However, nearly 66,000 eligible kidney transplant patients remained on the greater than 106,000 patient waitlist, and 6,427 (24.5%) donated kidneys went un-transplanted [1]. While the number of transplants continues to increase, the National Academies of Sciences, Engineering, and Medicine (NASEM) submitted a committee report to improve equity and accountability of the U.S. organ transplant system following their study on deceased donor organ procurement, allocation, and distribution [1]. The committee recommended four action areas to the current US organ transplant system to achieve equity in five years, with specific changes to kidney organ transplants [1]. As the NASEM committee report emphasizes, equity is still not achieved within the organ transplant system, and the importance of understanding that inequity regarding kidney transplantation cannot be underestimated as kidney transplants make up the majority of transplant and waitlisted patients.

Indications for kidney transplant

In the U. S., nearly 786,000 patients live with end-stage renal disease (ESRD) [2]. ESRD is the terminal stage of chronic kidney disease (CKD), defined as a glomerular filtration rate of less than 15 mL/min [3]. Patients with CKD have a reduced quality of life and premature mortality and are characterized by decreasing kidney function that can manifest as metabolic and electrolyte abnormality, metabolic acidosis, anemia, and symptoms, such as pericarditis, pleuritis, confusion, myoclonus, seizures, fluid overload, hypertension, peripheral neuropathy, restless leg, malnutrition, nausea, vomiting, etc. [3,4]. The cause of CKD and ESRD is kidney injury. Kidney injury can have multiple etiologies, with the most common being diabetes and hypertension [3].

Patients with ESRD require intervention, including recurrent dialysis and/or kidney transplant [3]. Patients are referred to a nephrologist to discuss the potential of kidney transplant when the estimated glomerular filtration rate (eGFR) is reduced to 30 or below, although patients who are asymptomatic may not initiate dialysis until eGFR is much lower [3]. While most patients with ESRD require dialysis, few may be eligible and undergo renal transplants. Kidney transplantation, while not a cure for renal disease, can increase a patient's quality of life and increase life expectancy [3]. To be eligible for a renal transplant, patients must join the waitlist maintained by United Network for Organ Sharing (UNOS). There are multiple factors that determine whether a patient receives a kidney transplant, including blood type, location, size of the patient compared to the donor, and in some cases, age and severity of the disease [5]. Recent changes have been made to the algorithm to determine organ allocation, including age matching of the donor and recipient (also named longevity matching) in 2014, the distance between donor and recipient instead of using region boundaries in 2019, and assigning priority for hard-to-match candidates who are highly sensitized like patients with higher antibody level due to previous transplants, blood transfusions or pregnancies in 2019 [4]. Not all patients who are placed on the transplant list can receive an organ depending on current health status, comorbidities, etc. However additional systemic factors also play a role in the access and ability of a patient to receive a renal transplant.

Kidney transplantation disparity

While the prevalence of ESRD has been stably increasing within the U.S. particular populations have an increased prevalence [3]. Minority populations, particularly Black and Hispanic populations, are more likely to have ESRD compared to the non-Hispanic white population (3.4 and 1.5 times higher, respectively) [2,3,6,7]. Additionally, ESRD is more prevalent in men and people over the age of 65 years old [3]. There is also a difference in prevalence regarding geographic location within the U.S., with those who live in the southern states, such as Arkansas, Louisiana, Texas, and Oklahoma, more likely to have ESRD compared to those living in the northeast [6].

Although minority populations have an increased prevalence of ESRD, their time spent on the waiting list continues to remain unequal. Published reports show that Black patients are less likely to be referred for transplant evaluation, are diagnosed with ESRD at lower eGFR, are delayed in transplantation registration, progress slower through the waiting list, and are ultimately less likely to receive a transplant compared to the non-Hispanic white population [6-10]. Continued criticism exists over the use of race-based kidney filtration equations that may overestimate filtration rate and misrepresent the severity of minority (specifically Blacks) kidney function, including what can be done to change it (e.g., creating new algorithms) [11].

In response to the racial and ethnic disparity in kidney transplantation protocol, changes have been made in three installments: 1) in 2003, the UNOS allocation policy was changed to eliminate priority based on HLA-B matching; 2) in 2014, UNOS changed the kidney allocation system to include first dialysis date as well as time entered onto the transplant list as the wait-time is one of the largest variables to determine kidney allocation; and 3) in December 2019, UNOS changed the region criteria to prioritize patients based on a 250-mile radius of procurement instead of using the fixed donation service areas created in the 1980s [12-14]. Studies conducted after the policy changes to determine the effectiveness in reducing racial/ethnic disparities showed that while there was a decrease in disparity, parity was not achieved regarding the transplant rate and time spent on the waitlist [13,15-17]. Few studies after 2014 and even fewer after 2019 have been conducted to determine if disparity has decreased in other areas after the new policy was installed. Within this context, we sought to examine the role of ethnicity/race along with geographic location, socioeconomic status, age, comorbidities, Kidney Donor Profile Index (KDPI), BMI, and gender in renal transplantation wait time disparities.

Although a recent analysis shows that the incidence of kidney transplantation for Black and white candidates is equivalent, it was hypothesized that ethnic and racial disparity still exists regarding the time spent on the Kidney Transplant Wait-List, which translates to a longer time spent receiving dialysis, associated with increased mortality, morbidity, and acute rejection of transplants, and decreased quality of life, for minority patients [9,18,19].

## Materials and methods

Data on all waitlisted candidates for donor kidney transplantation in the National Organ Procurement and Transplantation Network (OPTN) database listed on or before November 2020 were included in the analysis. Selection of censoring of data included using the OPTN advanced data report tool. Candidates listed for simultaneous kidney-pancreas transplants or other multi-organ transplants were excluded from the analysis. The data encompassed all adults, ages 18 years and older; pediatric candidates were excluded.

Candidate ethnicity was determined from the candidate race variable in the OPTN database and was grouped into seven subcategories: White, Black, Hispanic, Asian, American Indian/Alaskan Native, Pacific Islander, and Multiracial. Any candidate with a race/ethnicity variable listed as unknown was excluded from the OPTN advanced search. The waiting list population data included the time spent on the waiting list for each ethnicity/race in the following categories: <30 days; 30 to <90 days; 90 days to <6 months; six months to <1 year; one year to <2 years; two years to <3 years; three years to <5 years; and five or more years. Additional sub-categorical data were collected regarding the wait time of each ethnicity/race for potential covariates, such as gender, age, citizenship, the primary source of payment, region of transplant center, BMI, Kidney Donor Profile Index (KDPI), renal diagnosis, and presence/type of diabetes.

Time on the waiting list was calculated from the date of the listing, regardless of status, either active or inactive, to the time of the data collection. The waitlist candidates were then subcategorized by those who had been on the list for less than two years, two years and beyond, three years and beyond, and five years and beyond.

Data were analyzed using odds ratios and validated by Bonferroni-Holm’s corrected chi-square tests at confidence intervals of 95% to determine if there are statistically significant differences between transplant time spent on the waitlist and ethnicity, as well as age, diagnosis category, region of transplant center, and KDPI. All statistical analysis was performed using Microsoft Excel.

## Results

Characteristics of the sample

There were 91,830 patients included in the study; 35.3% (n=35,407) were listed as white, 31.6% (n=29.058) were listed as Black, 21.7% (n=19,883) were listed as Hispanic, 9.2% (n=8,419) were listed as Asian, 0.9% (n=851) were listed as American Indian/Alaskan Native, 0.6% (n=568) were listed as Pacific Islander, and 1.2% (n=1,063) were listed as multiracial. It is important to note that some patients may have been counted in multiple race and ethnic (i.e., Hispanic) categories. The median wait-list time was in the “1 year to <2 years” category; however, that time was not indicative of all patients' time on the waitlist, especially regarding race/ethnicity. 

Associations between race/ethnicity and waitlist wait times

A statistically significant association was found between race and waitlist waiting times. All non-white races examined, other than those identifying as multiracial, had statistically significant increased odds of remaining on the kidney transplant list greater than two years when compared to persons who identified as white. As can be seen in Table 1, the odds ratio and 95% confidence interval of each ethnicity/race, with the exception of multiracial, who remain on the waitlist at two, three, and five years are greater than one, which represents the odds of a white patient remaining on the waitlist at those same time intervals. 

**Table 1:** Associations between race/ethnicity and wait times and other covariates OR = Odds Ratio. OR_LB is the lower boundary odds ratio of the 95% confidence interval. OR_UB is the upper boundary odds ratio of the 95% confidence interval. Sig of 1 means that the odds ratio is statistically significantly different, with a p-value < 0.000001. Sig of 0 means that the odds ratio is not statistically significant.

		All Wait Times	Less than 2 Years versus 2+ Years Wait Times																								
Category	Subcategory	Bonferroni-Holms Corrected P-value	Bonferroni-Holms Corrected P-value	Odds Ratios vs. Whites for Waiting 2+ Years with 95% Confidence Intervals																							
				Black				Hispanic				Asian				Amer. Indian/Alaska Native				Pacific Islander				Multiracial			
				OR	OR_LB	OR_UB	Sig	OR	OR_LB	OR_UB	Sig	OR	OR_LB	OR_UB	Sig	OR	OR_LB	OR_UB	Sig	OR	OR_LB	OR_UB	Sig	OR	OR_LB	OR_UB	Sig
All Patients		0.00000	0.00000	1.384	1.342	1.428	1	1.365	1.319	1.414	1	1.513	1.444	1.586	1	1.278	1.116	1.463	1	1.38	1.17	1.627	1	1.115	0.987	1.26	0
		All Wait Times	Less than 3 Years versus 3+ Years Wait Times																								
Category	Subcategory	Bonferroni-Holms Corrected P-value	Bonferroni-Holms Corrected P-value	Odds Ratios vs. Whites for Waiting 3+ Years with 95% Confidence Intervals																							
				Black				Hispanic				Asian				Amer. Indian/Alaska Native				Pacific Islander				Multiracial			
				OR	OR_LB	OR_UB	Sig	OR	OR_LB	OR_UB	Sig	OR	OR_LB	OR_UB	Sig	OR	OR_LB	OR_UB	Sig	OR	OR_LB	OR_UB	Sig	OR	OR_LB	OR_UB	Sig
All Patients		0.000000	0.00000	1.447	1.399	1.496	1	1.403	1.351	1.457	1	1.551	1.476	1.63	1	1.312	1.135	1.516	1	1.388	1.166	1.652	1	1.057	0.923	1.21	0
		All Wait Times	Less than 5 Years versus 5+ Years Wait Times																								
Category	Subcategory	Bonferroni-Holms Corrected P-value	Bonferroni-Holms Corrected P-value	Odds Ratios vs. Whites for Waiting 5+ Years with 95% Confidence Intervals																							
				Black				Hispanic				Asian				Amer. Indian/Alaska Native				Pacific Islander				Multiracial			
				OR	OR_LB	OR_UB	Sig	OR	OR_LB	OR_UB	Sig	OR	OR_LB	OR_UB	Sig	OR	OR_LB	OR_UB	Sig	OR	OR_LB	OR_UB	Sig	OR	OR_LB	OR_UB	Sig
All Patients		0.00000	0.00000	1.528	1.461	1.599	1	1.448	1.376	1.523	1	1.638	1.536	1.747	1	1.367	1.129	1.656	1	1.78	1.438	2.203	1	0.939	0.771	1.144	0

**Figure 1:**
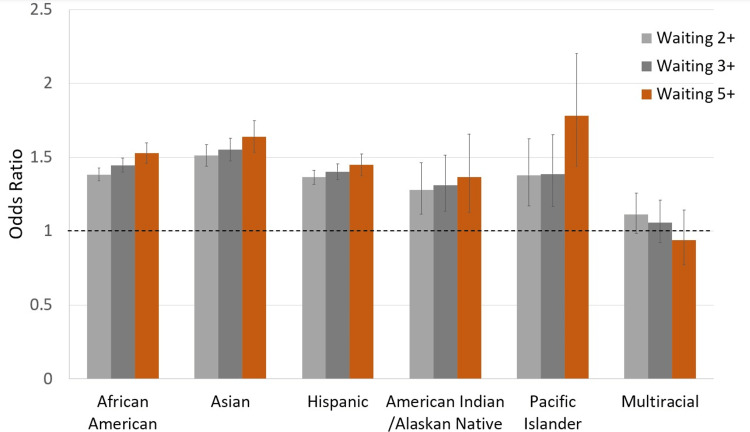
Odds ratio comparisons of whites to other racial/ethnic groups for kidney transplants in years at 95% confidence intervals 2+ refers to two or more years; 3+ refers to three or more years; 5+ refers to five or more years.

Figure 1 also demonstrates visually the odds ratio and 95% confidence interval of each ethnicity/race versus the odds of a white patient remaining on the waitlist at those same time intervals.

Table 2 reports the odds ratio and 95% confidence interval of each race/ethnicity who remain on the waitlist for two or more years remains statistically significant for all subcategories that were included in the study, indicating a general effect not being driven by any one factor. The size of this effect, however, may differ when analyzing the subcategories of data and it should be noted that the effect size was insufficiently large for the relatively small count of candidates who identified as American Indian/Alaskan Native, Pacific Islander, and multiracial to remain statistically significant. Additionally, the sub-categorical data was not analyzed for wait-list times greater than three years.

**Table 2:** Associations between race/ethnicity and wait times and other covariates OR = Odds Ratio. OR_LB is the lower boundary odds ratio of the 95% confidence interval. OR_UB is the upper boundary odds ratio of the 95% confidence interval. Sig of 1 means that the odds ratio is statistically significantly different, with a p-value < 0.000001. Sig of 0 means that the odds ratio is not statistically significant.

		All Wait Times	Less than 2 Years versus 2+ Years Wait Times																								
Category	Subcategory	Bonferroni-Holms Corrected P-value	Bonferroni-Holms Corrected P-value	Odds Ratios vs. Whites for Waiting 2+ Years with 95% Confidence Intervals																							
				Black				Hispanic				Asian				Amer. Indian/Alaska Native				Pacific Islander				Multiracial			
				OR	OR_LB	OR_UB	Sig	OR	OR_LB	OR_UB	Sig	OR	OR_LB	OR_UB	Sig	OR	OR_LB	OR_UB	Sig	OR	OR_LB	OR_UB	Sig	OR	OR_LB	OR_UB	Sig
All Patients		0	0	1.384	1.342	1.428	1	1.365	1.319	1.414	1	1.513	1.444	1.586	1	1.278	1.116	1.463	1	1.38	1.17	1.627	1	1.115	0.987	1.26	0
Gender	Male	0	0	1.407	1.352	1.463	1	1.393	1.333	1.454	1	1.484	1.397	1.576	1	1.344	1.121	1.611	1	1.34	1.071	1.677	1	1.215	1.035	1.427	1
	Female	0	0	1.348	1.283	1.418	1	1.319	1.244	1.397	1	1.554	1.442	1.674	1	1.194	0.973	1.465	0	1.421	1.114	1.812	1	0.989	0.818	1.195	0
Age	18-34 Years	0.00021	0.00019	1.357	1.207	1.526	1	1.296	1.154	1.455	1	1.319	1.097	1.586	1	1.179	0.725	1.918	0	1.126	0.615	2.062	0	0.921	0.641	1.321	0
	35-49 Years	0	0	1.481	1.383	1.585	1	1.465	1.359	1.58	1	1.458	1.316	1.616	1	1.188	0.904	1.562	0	1.518	1.106	2.083	1	1.14	0.901	1.443	0
	50-64 Years	0	0	1.418	1.352	1.486	1	1.438	1.363	1.517	1	1.526	1.42	1.64	1	1.391	1.133	1.709	1	1.401	1.101	1.783	1	1.373	1.129	1.671	1
	65 +	0	0	1.406	1.322	1.496	1	1.587	1.47	1.714	1	1.661	1.52	1.814	1	1.569	1.169	2.106	1	1.455	0.971	2.179	0	1.219	0.912	1.63	0
Citizenship	U.S. Citizen	0	0	1.376	1.334	1.42	1	1.283	1.233	1.335	1	1.455	1.381	1.532	1	1.285	1.12	1.474	1	1.344	1.122	1.61	1	1.116	0.985	1.263	0
	Non-U.S. Citizen	na	0.2049	1.086	0.881	1.339	0	1.178	0.995	1.395	0	1.33	1.104	1.603	1	1.041	0.298	3.635	0	1.276	0.815	1.996	0	0.603	0.282	1.289	0
Primary Source of Payment	Private insurance	0	0	1.38	1.316	1.446	1	1.222	1.157	1.291	1	1.403	1.314	1.498	1	1.039	0.824	1.309	0	1.335	1.04	1.714	1	0.998	0.836	1.191	0
	All Public insurance	0	0	1.41	1.352	1.47	1	1.475	1.409	1.545	1	1.597	1.492	1.709	1	1.462	1.233	1.732	1	1.435	1.15	1.792	1	1.192	1.005	1.414	1
Region of Center	Region 1	na	0.01834	1.319	1.139	1.528	1	1.078	0.911	1.276	0	1.248	0.991	1.572	0	0.778	0.276	2.191	0	na	na	na	na	0.718	0.383	1.344	0
	Region 2	na	0	1.456	1.348	1.573	1	1.061	0.929	1.213	0	1.089	0.942	1.258	0	1.336	0.555	3.215	0	0.89	0.316	2.506	0	0.89	0.55	1.441	0
	Region 3	na	0	1.507	1.391	1.632	1	0.995	0.878	1.128	0	1.292	1.044	1.598	1	1.177	0.668	2.074	0	0.963	0.342	2.711	0	0.391	0.211	0.724	1
	Region 4	na	0	1.496	1.333	1.678	1	1.234	1.109	1.372	1	1.192	0.967	1.47	0	0.789	0.473	1.317	0	0.689	0.302	1.571	0	0.63	0.346	1.145	0
	Region 5	0	0	1.493	1.349	1.651	1	1.247	1.163	1.336	1	1.371	1.262	1.489	1	1.226	0.99	1.517	0	1.355	1.088	1.689	1	0.97	0.783	1.202	0
	Region 6	0.04944	0.10018	1.14	0.857	1.517	0	0.782	0.596	1.024	0	1.2	0.972	1.483	0	0.908	0.502	1.641	0	0.843	0.567	1.254	0	1.921	1.212	3.045	1
	Region 7	na	0.00077	1.332	1.191	1.489	1	1.047	0.907	1.209	0	1.223	1.022	1.463	1	1.096	0.797	1.508	0	1.291	0.322	5.171	0	1.537	1.014	2.329	1
	Region 8	na	0.00018	1.552	1.313	1.836	1	1.468	1.197	1.8	1	1.07	0.776	1.478	0	1.209	0.624	2.344	0	1.182	0.333	4.201	0	1.273	0.777	2.085	0
	Region 9	na	0	1.55	1.388	1.731	1	1.343	1.183	1.526	1	1.395	1.198	1.625	1	1.341	0.628	2.865	0	2.49	0.455	13.624	0	0.639	0.366	1.118	0
	Region 10	na	0	1.554	1.382	1.747	1	0.916	0.686	1.223	0	0.816	0.589	1.131	0	1.387	0.546	3.525	0	1.156	0.326	4.106	0	1.885	1.065	3.337	1
	Region 11	na	0.01812	1.213	1.112	1.325	1	1.02	0.803	1.295	0	0.915	0.698	1.199	0	1.015	0.563	1.827	0	0.966	0.282	3.307	0	1.294	0.924	1.813	0
	All Regions Excluding 5 & 6	0	0	1.422	1.376	1.47	1	1.142	1.092	1.195	1	1.246	1.167	1.331	1	1.074	0.888	1.298	0	0.857	0.579	1.267	0	0.984	0.838	1.155	0
BMI	18.5-<25	0	0	1.357	1.264	1.457	1	1.271	1.178	1.37	1	1.483	1.363	1.614	1	1.713	1.205	2.435	1	1.052	0.63	1.756	0	0.967	0.728	1.286	0
	25-<30	0	0	1.404	1.325	1.488	1	1.445	1.359	1.537	1	1.501	1.381	1.632	1	1.062	0.833	1.355	0	1.337	0.969	1.844	0	1.015	0.795	1.296	0
	30-<35	0	0	1.388	1.306	1.475	1	1.34	1.251	1.436	1	1.525	1.363	1.707	1	1.269	0.994	1.62	0	1.548	1.144	2.095	1	1.143	0.903	1.446	0
	35-<50	0	0	1.325	1.229	1.428	1	1.313	1.191	1.447	1	1.407	1.151	1.719	1	1.523	1.105	2.1	1	1.254	0.861	1.828	0	1.266	0.935	1.714	0
KDPI	<86	0	0	1.384	1.342	1.428	1	1.365	1.319	1.414	1	1.513	1.444	1.586	1	1.278	1.116	1.463	1	1.38	1.17	1.627	1	1.115	0.987	1.26	0
	86+	0	0	1.373	1.318	1.431	1	1.379	1.318	1.443	1	1.614	1.519	1.714	1	1.307	1.114	1.535	1	1.642	1.344	2.005	1	1.099	0.948	1.276	0
Diagnosis	Diabetes	0	0	1.36	1.287	1.436	1	1.549	1.464	1.639	1	1.693	1.567	1.828	1	1.535	1.274	1.849	1	1.492	1.189	1.873	1	1.261	1.031	1.543	1
	Glomerular Disease	0	0	1.426	1.317	1.544	1	1.267	1.16	1.383	1	1.44	1.299	1.597	1	1.122	0.784	1.606	0	1.152	0.746	1.778	0	0.928	0.693	1.243	0
	Hypertensive Nephrosclerosis	0	0	1.402	1.305	1.506	1	1.304	1.187	1.431	1	1.5	1.331	1.691	1	1.259	0.807	1.965	0	1.497	0.934	2.397	0	1.281	0.918	1.787	0
	Polycystic Kidneys	na	0	1.492	1.302	1.709	1	1.336	1.154	1.547	1	1.203	0.961	1.506	0	1.125	0.558	2.269	0	2.17	0.771	6.109	0	0.89	0.533	1.487	0
	Retransplant/Graft Failure	na	0	1.417	1.263	1.59	1	1.34	1.154	1.555	1	1.511	1.252	1.824	1	1.587	0.874	2.882	0	1.276	0.54	3.014	0	1.206	0.812	1.792	0
	Tubular and Interstitial Diseases	na	0.23662	1.043	0.855	1.272	0	0.963	0.778	1.192	0	1.123	0.828	1.522	0	0.332	0.132	0.835	1	0.601	0.175	2.059	0	1.191	0.592	2.399	0
Diabetes	No	0	0	1.392	1.336	1.451	1	1.257	1.197	1.32	1	1.42	1.331	1.514	1	1.122	0.898	1.401	0	1.495	1.146	1.95	1	1.122	0.95	1.326	0
	Type I	na	0.269	1.211	1.033	1.419	1	1.242	1.032	1.495	1	1.484	0.987	2.232	0	0.606	0.189	1.939	0	0.909	0.217	3.814	0	0.679	0.351	1.314	0
	Type II	0	0	1.399	1.33	1.473	1	1.508	1.429	1.592	1	1.653	1.538	1.776	1	1.471	1.232	1.755	1	1.358	1.095	1.684	1	1.157	0.955	1.401	0

Although candidates who identified as Black, Hispanic, Asian, American Indian/Alaskan Native, or Pacific Islander maintained higher odds of remaining on the waitlist than candidates identifying as white, each race/ethnicity had varying odds. The odds of a candidate categorized as Black waiting two or more years for a transplant is 1.38 times that of a candidate categorized as White, with a 95% confidence interval of (1.34, 1.43). The odds of a candidate categorized as Hispanic waiting two or more years for a transplant is 1.37 times that of a patient categorized as white, with a 95% confidence interval of (1.32, 1.41). The odds of a candidate categorized as Asian waiting two or more years for a transplant is 1.51 times that of a candidate categorized as white, with a 95% confidence interval of (1.44, 1.59). The odds of a candidate categorized as American Indian or Alaska Native waiting two or more years for a transplant is 1.28 times that of a candidate categorized as white, with a 95% confidence interval of (1.12, 1.46). The odds of a candidate categorized as a Pacific Islander waiting two or more years for a transplant is 1.38 times that of a candidate categorized as white, with a 95% confidence interval of (1.17, 1.63). The odds of a candidate categorized as multiracial waiting two or more years for a transplant is 1.12 times that of a candidate categorized as white, with a 95% confidence interval of (0.99, 1.26). Notably, this is not statistically significant, indicating that this is not contributing to the overall statistically significant association between race and wait time.

## Discussion

As the prevalence of ESRD increases and the number of kidneys required for kidney transplantation increases, the current organ distribution system will need to continually undergo revision to provide patients with the best quality of life and prognosis. While the number of successful transplants continues to increase, thousands of people remain on the transplant list, with kidney transplant patients making up the majority. Although it has been widely documented that ESRD disproportionately affects minority, elderly, and southern patients, the current organ distribution system has historically been unable to adequately provide for those populations. Policy changes over the past two decades have been directed at improving the areas that have created disparity, such as HLA matching, incorporating first-time requiring dialysis as a parameter to determine kidney allocation, and updating kidney distribution based on distance from the geographic location of organ procurement [12-14]. Reassessment of patients’ time spent on the waitlist after these changes have been made is a good indicator of the success of these policies, as increasing time on the waitlist and a lengthened time requiring dialysis increases morbidity, mortality, and acute organ rejection and decreases patients’ quality of life [18]. Although prevention of progression to ESRD is the ideal goal, for a patient who requires a placement on the kidney transplant list the outcome should not be affected by their immutable characteristics.

Ethnicity as a major factor in time spent on the kidney transplant waitlist

In this study, ethnic disparities persisted for non-white individuals receiving treatment for end-stage kidney disease, specifically in the context of time spent on the waitlist for a kidney transplant. These differences persisted even up to a five-year wait time. Despite changes in organ allocation policy, minorities waitlisted for renal transplantation remain disadvantaged compared with patients who identify as white. Although Black and Hispanic/Latinx populations remain on the wait time longer than white populations, Asian American patients were found to be most disadvantaged. Asian American patients had the greatest odds of remaining on the waitlist greater than two years and greater than three years in comparison to white patients: 1.51 times more likely to remain on the list after two years and 1.55 times more likely to remain on the list after three years than that of a patient who identified as white.

Additionally, patients that identified as Pacific Islanders were 1.78 times more likely to remain on the list after five years than that of a patient who identified as white, the highest odds among all ethnic/racial groups on the waitlist for 5+ years. Previous studies assessing the 2014 policy changes on the kidney allocation system (KAS) found that the number of Asian patients on the waitlist decreased but continued to have the highest wait-listing rate, the rate at which added to the list [17]. In 2019, Asians made up 8.5% of the organ transplant waitlist, but only 24.7% of those patients received transplants, whereas 48.8% of white patients on the transplant list received transplants [20]. Few studies exist to determine the reason for this disparity. A qualitative study of organ donation-related attitudes and beliefs of three Asian ethnic groups located in the greater Philadelphia metropolitan area: Chinese, Filipino, and Vietnamese Americans found that Asian communities had beliefs that may limit the relatability of U.S. organ donation and transplant campaigns, especially among older populations [21]. It has been postulated that Asian Americans would benefit more from a micro-targeted culturally competent family approach, as Asian Americans were mainly supportive of organ donation and transplants if kept in the family [21]. Additionally, nearly one-third of the Asian participants interviewed during that study cited religious beliefs and the idea that they needed to remain whole with all of their organs as a barrier to organ donation [21]. While this research helped elucidate opinions on donation, it does not provide insight into barriers of Asian patients who remain on the kidney transplant list. More research needs to be conducted to better understand why Asian patients have higher odds of remaining on the transplant list longer than white Americans.

Pacific Islanders make up 0.6% of US Transplant waitlist candidates but only 0.2% of the US population [22]. While the research regarding Asian American transplant patients is limited, even fewer studies exist that analyze the views and opinions of Pacific Islanders. Additionally, only 25% of Pacific Islanders on the waitlist received a transplant versus 48.8% of the white population [22]. Native Hawaiians, a segment of the Pacific Islander population, are more likely to be diagnosed with diabetes, cardiovascular disease, higher obesity rates, and more likely to be diagnosed with chronic liver disease when compared to white Hawaiians [22]. Limited research on this population leaves many questions as to why at five years, they are 1.78 times more likely to remain on the kidney transplant list compared to patients that identify as White. More research is required to explain this finding. Recent articles researching changes in waitlist times after the kidney allocation system policies were updated were unable to determine the effects on the Native Hawaiian Pacific Islander population [17].

Study limitations

While this study included a large sample and an up-to-date database (including data collected one year after major policy changes made in 2019 by UNOS) and findings may be considered generalizable to the population's studies, it has its limitations. The results cannot define a change to the waitlist overtime; inactive patients may have more heavily impacted smaller minority populations, inequality between the geographic location of candidates could not be assessed, socioeconomic status was not easily quantified, and the population is only representative of the adult patient population with ESRD. Associations between race/ethnicity and odds of remaining on the waitlist as compared to candidates that identify as white cannot define changes to the waitlist and time spent on the waitlist post versus pre-policy change completed in 2019, as there are no previous odds ratio studies completed before the policy change. These findings can only describe an existing inequality/disparity that exists at the time of data gathering.

Additionally, as status seven candidates were not specifically excluded from the data, some of the candidates may remain on the waitlist even if an organ was offered. A status seven candidate is a candidate that is temporarily unsuitable for transplant due to reasons such as the candidate being too sick for transplant, lacking insurance, having medical, surgical, or psychosocial contraindications, or the patient chooses not to receive the organ [23]. Zhang et al. concluded that while racial disparities continue to exist on the waitlist, the racial disparity reduction seen after the kidney allocation system was updated in 2014 was due to a “steeper decline in inactive waitlisting among minorities and a greater proportion of actively waitlisted minority patient” [17]. Although the data were collected after a large amount of inactive or status 7 candidates were removed from the list due to changes in the kidney allocation system, the odds ratio may still be slightly affected by the limited numbers of candidates from some ethnic/racial groups [17]. Furthermore, it should be noted that while people were removed from the list due to inactive status, the literature shows that candidates who identify as white are more likely to move from inactive to active status, allowing them to receive a transplant than patients who identify as an ethnic/racial minority group [16].

Another limitation is the inability to further evaluate and analyze the geographic location of the patient. Candidate data from all 11 regions within the U.S. were collected and analyzed as a covariate, and while the odds ratio for some ethnic/racial minorities remained statistically significant throughout most regions, such as candidates that identified as Black who had increased odds regardless of location, with the exception of region 6, which includes Alaska, Hawaii, Idaho, Montana, Oregon, and Washington, smaller groups such as Asian American, Native Hawaiian and Pacific Islander, and American Indian Alaskan native did not remain statistically significant [24]. This may be due to the limited number of candidates in the specific regions or may indicate that there is a regional disparity that deserves greater evaluation. Previous data that analyzed the location and geographic disparity caused by the organ allocation system also utilized Designated Service Areas (DSA), which may not be able to be directly compared with regions of OPTN [25].

This study also neglected to account for data that can identify the socioeconomic status of the candidates on the waitlist. Patzer and McClellan found that low socioeconomic status is correlated with kidney disease progression and access to kidney transplantation [19]. In addition, low SES is further associated with an increased incidence of CKD, progression to end-stage renal disease, inadequate dialysis treatment, reduced access to kidney transplantation, and poor health outcomes [19]. While this study evaluates insurance type, it is not the best indicator of socioeconomic status. Previous studies have shown that the insurance type (e.g., private, Medicaid, or no insurance) of the candidates on the waitlist has been a barrier to transplant, can exacerbate disparity, or lead to worse survival outcomes [26,27]. In this study, the type of insurance did not seem to impact the odds, as the increased odds remained statistically significant regardless of private or public insurance. Due to limitations in data sets, further subcategorization and patients with no insurance were not specified.

Although previous studies have shown similar racial/ethnic disparities in pediatric patients with ESRD, this population was not considered in this study [28]. This study was limited to adult candidates and did not analyze pediatric patients under age 18, and further evaluation is warranted.

Implications for future research 

After the data in this study were analyzed, OPTN made updates to race and ethnicity reporting in January 2022, allowing race and ethnicity to be reported instead of categorizing individuals based on ethnicity. The change was made because some individuals identify as two or more ethnicities based on the previous categorization, such as Hispanics that identify as white. Ethnicity misclassification that was inherent in the data collected by OPTN may have influenced the outcome of this study, and further research should be done to determine if changes to data reporting have impacted the wait time.

## Conclusions

Misinformation and lack of emphasis on change of what was previously considered race-based medicine may still impact individuals who identify as a minority or a non-white American. As racial and ethnic disparities have been seen in waitlist time for organ transplantation, including kidney transplantation, this research highlights health disparities in nephrology, or more specifically for individuals who are receiving treatment for end-stage kidney disease. Overall, a statistically significant association was found between race and waitlist waiting times. All non-white candidates examined, other than those identifying as multiracial, had statistically significant increased odds of remaining on the kidney transplant list greater than two years when compared to persons who identified as white. Asian American candidates had the greatest odds of remaining on the waitlist greater than two years in comparison to white candidates: 1.51 times more likely to remain on the list after two years than a candidate who identified as white. African American and Pacific Islander candidates had odds of remaining on the transplant waitlist greater than two years which was slightly lower than that of Asian Americans in comparison to white patients: 1.38 times that of candidates who identified as white. Among odds ratios that are statistically different from white candidates, Hispanic candidates, followed by Native American and Alaskan candidates, had the smallest odds of remaining on the waitlist greater than two years in comparison to white candidates: 1.37 times and 1.28 times that of candidates that identified as white, respectively. Recognizing these disparities may prompt researchers to further investigate the causes of this disparity and assist in changing policy to close the gap in future transplantation practice. Further research should be conducted on the causes of these disparities in time spent on the waitlist, cultural restrictions in organ donation, racial differences in parameters for organ match, as well as the possible role of institutionalized racism in health care practices and practitioners.
